# Bibliometric and visualized analysis of the application of artificial intelligence in stroke

**DOI:** 10.3389/fnins.2024.1411538

**Published:** 2024-09-11

**Authors:** Fangyuan Xu, Ziliang Dai, Yu Ye, Peijia Hu, Hongliang Cheng

**Affiliations:** ^1^The First Clinical Medical School, Anhui University of Chinese Medicine, Hefei, China; ^2^Department of Rehabilitation Medicine, The Second Hospital of Wuhan Iron and Steel (Group) Corp., Wuhan, China; ^3^The Second Clinical Medical School, Anhui University of Chinese Medicine, Hefei, China; ^4^The Second Affiliated Hospital of Anhui University of Chinese Medicine, Hefei, China; ^5^Anhui Province Key Laboratory of Meridian Viscera Correlationship, Hefei, China

**Keywords:** artificial intelligence, stroke, machine learning, bibliometric analysis, VOSviewer, CiteSpace

## Abstract

**Background:**

Stroke stands as a prominent cause of mortality and disability worldwide, posing a major public health concern. Recent years have witnessed rapid advancements in artificial intelligence (AI). Studies have explored the utilization of AI in imaging analysis, assistive rehabilitation, treatment, clinical decision-making, and outcome and risk prediction concerning stroke. However, there is still a lack of systematic bibliometric analysis to discern the current research status, hotspots, and possible future development trends of AI applications in stroke.

**Methods:**

The publications on the application of AI in stroke were retrieved from the Web of Science Core Collection, spanning 2004–2024. Only articles or reviews published in English were included in this study. Subsequently, a manual screening process was employed to eliminate literature not pertinent to the topic. Visualization diagrams for comprehensive and in-depth analysis of the included literature were generated using CiteSpace, VOSviewer, and Charticulator.

**Results:**

This bibliometric analysis included a total of 2,447 papers, and the annual publication volume shows a notable upward trajectory. The most prolific authors, countries, and institutions are Dukelow, Sean P., China, and the University of Calgary, respectively, making significant contributions to the advancement of this field. Notably, stable collaborative networks among authors and institutions have formed. Through clustering and citation burst analysis of keywords and references, the current research hotspots have been identified, including machine learning, deep learning, and AI applications in stroke rehabilitation and imaging for early diagnosis. Moreover, emerging research trends focus on machine learning as well as stroke outcomes and risk prediction.

**Conclusion:**

This study provides a comprehensive and in-depth analysis of the literature regarding AI in stroke, facilitating a rapid comprehension of the development status, cooperative networks, and research priorities within the field. Furthermore, our analysis may provide a certain reference and guidance for future research endeavors.

## 1 Introduction

Stroke stands as a prominent cause of death and disability worldwide, presenting a significant public health threat. In 2016, there were 13.7 million new stroke incidents worldwide, with approximately 87% being ischemic strokes (IS) (Saini et al., 2021). Through the analysis of data from 1,599 hospitals across China in 2020, it was found that IS comprised 81.9% of the total stroke cases, while intracerebral hemorrhage and subarachnoid hemorrhage strokes accounted for 14.9 and 3.1%, respectively (Tu et al., 2023). A study predicted that mortalities of stroke worldwide will surge from 6.6 million in 2020 to 9.7 million by 2050 (Feigin et al., 2023). The incidence of stroke and its sequelae severely impacts patients’ quality of life and imposes a substantial economic burden. It was estimated that the annual direct costs (such as treatment and rehabilitation) and indirect costs (productivity loss) associated with global stroke exceed $891 billion (Feigin et al., 2023). Therefore, it is necessary to explore effective approaches to enhance diagnostic accuracy and efficiency, guide clinical treatment, rehabilitation, and acute care methods, and predict stroke prognosis.

Artificial intelligence (AI), a branch within computer science, emulates thinking processes, learning abilities, and knowledge management (Sak and Suchodolska, 2021). Its integration into healthcare has experienced rapid growth in recent years, with numerous research teams exploring its application in stroke imaging analysis, assistive rehabilitation, treatment, clinical decision-making, as well as outcome and risk prediction for stroke (Heo et al., 2019; Mouridsen et al., 2020; Yedavalli et al., 2021; Lip et al., 2022; Liu et al., 2022). Studies have demonstrated that AI facilitates decreasing inter-rater divergence, thereby enhancing the standardization of stroke patient assessment. Furthermore, it enables the rapid identification of valuable imaging data for aiding in the selection of treatment schemes and bolstering clinical decision-making (Bivard et al., 2020; Akay et al., 2023). Nonetheless, a systematic bibliometric analysis is still lacking to identify the current research status, hotspots, and potential future trends in AI applications for stroke.

Bibliometric analysis utilizes mathematical and statistical methods to conduct scientific quantitative analysis of literature within a certain field. This approach enables the examination of collaborative relationships among authors, institutions, and countries, thus obtaining directions for further cooperation and academic communication. Through a thorough analysis of keywords and references extracted from pertinent literature, we can better comprehend the knowledge foundation and development context regarding the application of AI in stroke. Commonly utilized software for bibliometric analysis, such as VOSviewer and CiteSpace, generates visualization graphs to elucidate research hotspots and trends more clearly and intuitively. Consequently, we perform this bibliometric analysis of relevant publications spanning 2004–2024, aiming to provide useful information for subsequent studies.

## 2 Materials and methods

### 2.1 Literature source and data retrieval methods

The Web of Science Core Collection (WoSCC) is a crucial database for accessing global academic information, comprising a substantial collection of influential and high-quality publications. We selected this database for our analysis due to its ability to extensively retrieve relevant literature and its widespread use in bibliometric analysis (Jiang et al., 2023; Song et al., 2024; Yang et al., 2024). In this study, we conducted a comprehensive retrieval of the WoSCC database from January 1, 2004 to March 1, 2024 to identify pertinent literature on the application of AI in stroke. The search formula was TS = (“artificial intelligence” OR “AI” OR “machine intelligence” OR “computational intelligence” OR “robot*” OR “computer reasoning” OR “computer vision system” OR “neural network*” OR “machine learning” OR “deep learning” OR “natural language processing”) AND TS = (“stroke” OR “cerebrovascular accident” OR “CVA” OR “cerebrovascular apoplexy” OR “brain vascular accident” OR “cerebrovascular stroke” OR “cerebral stroke” OR “apoplexy” OR “acute stroke” OR “acute cerebrovascular accident”). We included reviews and articles in our study, restricting the language of publications to English. After the screening of the titles and abstracts of the retrieved literature, incomplete, duplicate, and irrelevant studies were excluded. Subsequently, the remaining publications were exported with full records and cited references in TXT form.

### 2.2 Data analysis

**Figure 1:**
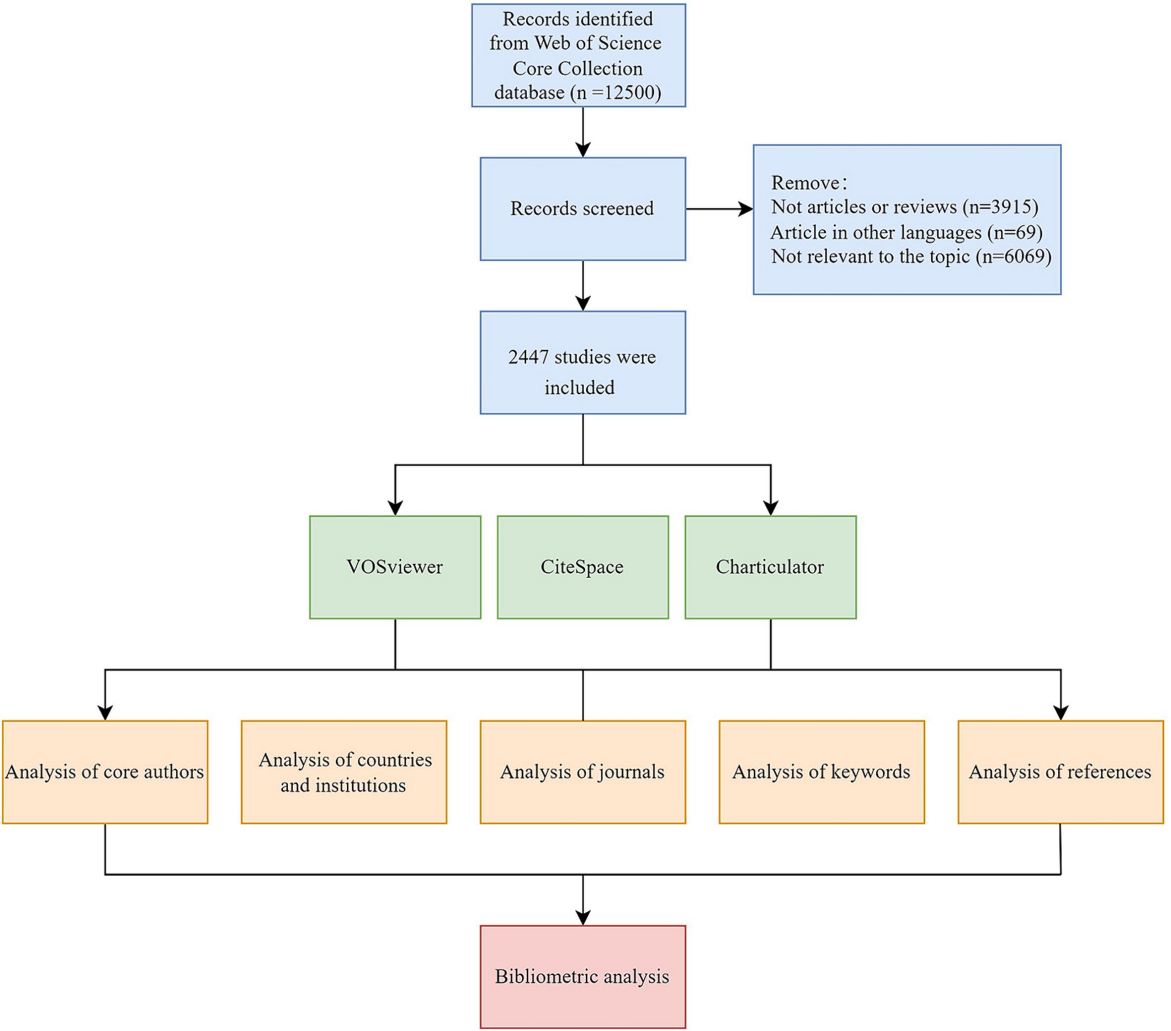
The flow chart for screening literature and analysis.

This bibliometric analysis primarily employed CiteSpace 6.3.R1 (Chen, 2006) and VOSviewer V1.6.19 tools (van Eck and Waltman, 2010) to analyze pertinent information and references from the included literature, thus identifying research foci and frontiers. VOSviewer generates visualization network maps of journals and keywords, along with illustrating collaborative relationships among core authors and institutions. Node size corresponds to publication frequency, while the lines depict the intensity of the relationship between nodes. Additionally, a chord diagram was created using Charticulator to visualize the collaboration between countries or regions. CiteSpace was applied to conduct the dual-map overlay of journals and clustering of references. Besides, it generated citation bursts of keywords and references, providing valuable information for exploring the development status and trends of research. The flow chart for screening literature and analysis is displayed in Figure 1.

## 3 Results

### 3.1 Analysis of publication outputs

**Figure 2:**
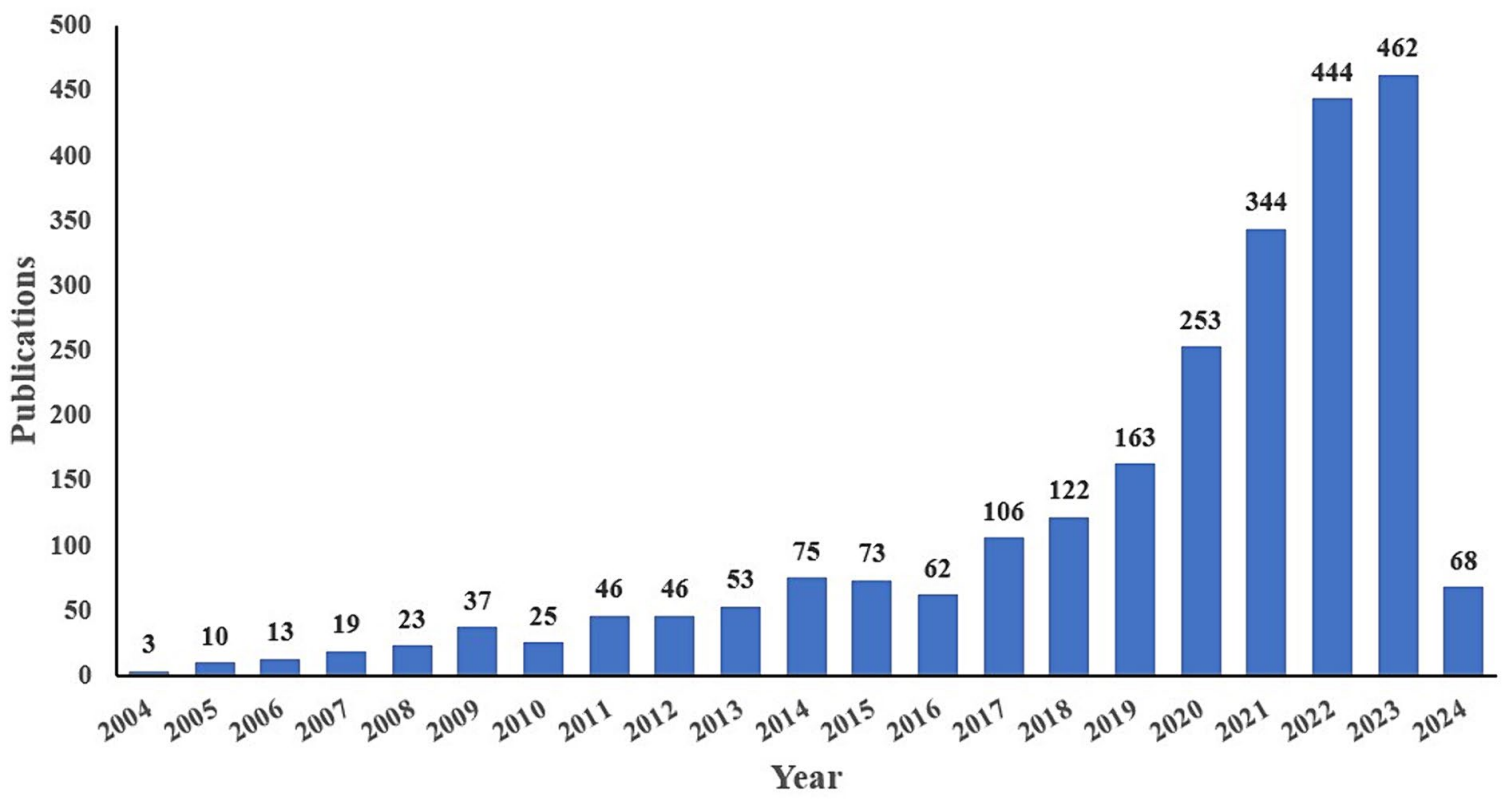
The annual publication trend map of the included literature.

Through the search and screening of pertinent literature spanning from 2004 to 2024, a total of 2,447 publications are included, comprising 2,215 articles and 232 reviews. The annual publication trend regarding AI application in stroke is depicted in Figure 2, revealing a consistent increase over time. This suggests a growing scholarly interest in applying AI to stroke research. Notably, there was a peak of 462 articles published in 2023 and 68 articles published in the first 2 months of 2024.

### 3.2 Analysis of authors

**Figure 3:**
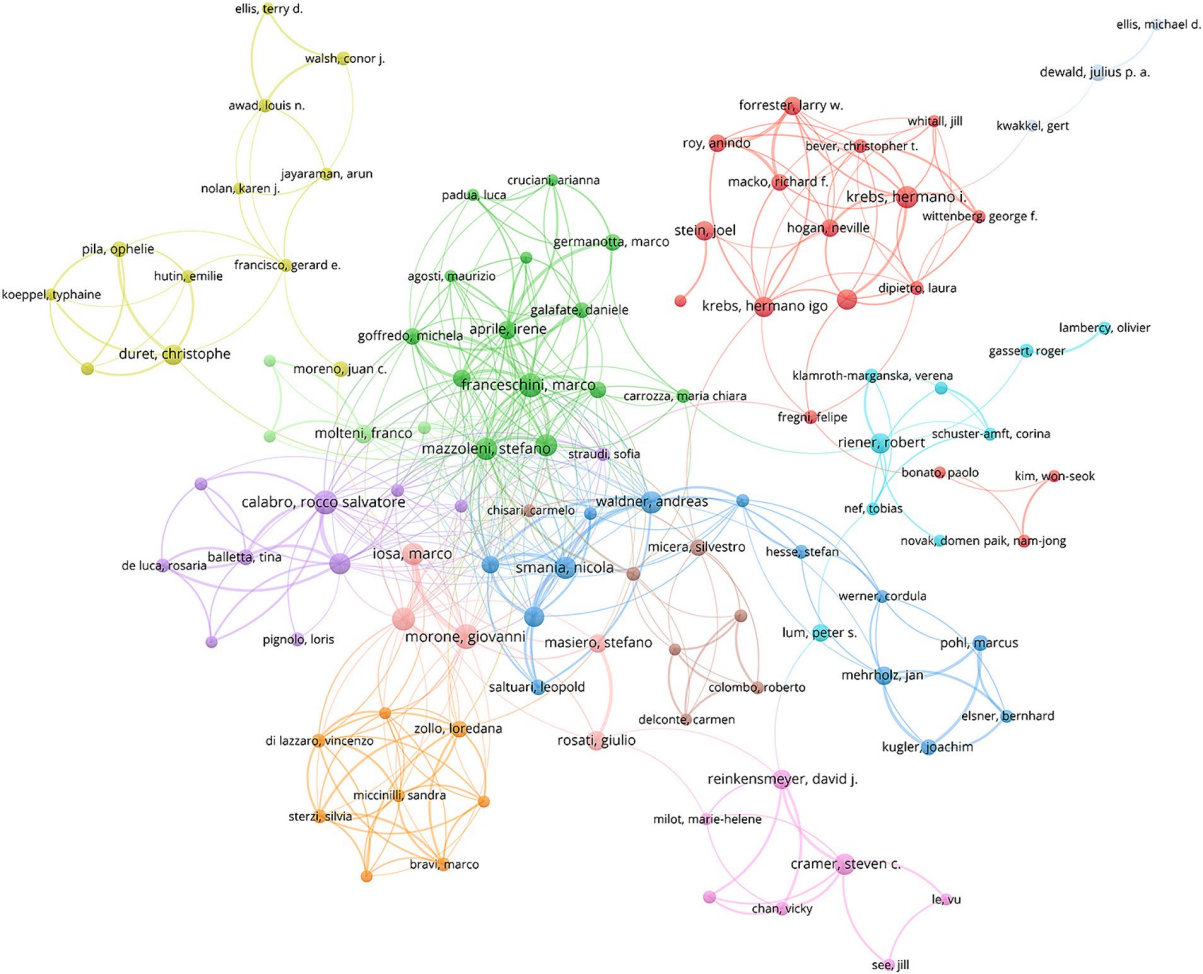
The collaboration network visualization map of the core authors.

A total of 11,883 authors have contributed to the literature concerning the application of AI to stroke. According to Price’s law, the minimum publication volume of core authors in a certain field is m = 0.749× $\sqrt{n_{\text{max}}}$ , where *n*_max_ represents the publication counts of the most prolific author. Therefore, a core author should have at least published five papers. Figure 3 illustrates the collaboration network map of core authors. It can be seen that several stable author collaboration groups have been formed. Table 1 lists the top 10 most productive authors concerning the application of AI to stroke. Notably, Dukelow, Sean P. leads with 35 articles. His studies primarily focused on using AI to assess proprioceptive impairment and visuomotor adaptation impairment after stroke, as well as promoting stroke rehabilitation through robotic interventions (Dukelow, 2017; Keeling et al., 2021; Moore et al., 2022; Chilvers et al., 2023; Hossain et al., 2023). Additionally, Scott, Stephen H. gained a relatively higher average citation, indicating his publications are influential and recognized by scholars.

**Table 1:** The top 10 authors about the application of AI in stroke.

Rank	Author	Documents	Citations	Average citation
1	Dukelow, Sean P.	35	1,335	38.14
2	Wu, ching-yi	30	766	25.53
3	Scott, Stephen H.	26	1,218	46.85
4	Lin, Keh-chung	26	776	29.85
5	Morone, Giovanni	21	707	33.67
6	Franceschini, Marco	20	490	24.50
7	Fiehler, Jens	20	132	6.60
8	Calabro, Rocco Salvatore	20	685	34.25
9	Suri, Jasjit S	19	573	30.16
10	Saba, luca	19	573	30.16

### 3.3 Analysis of countries/regions and institutions

**Figure 4:**
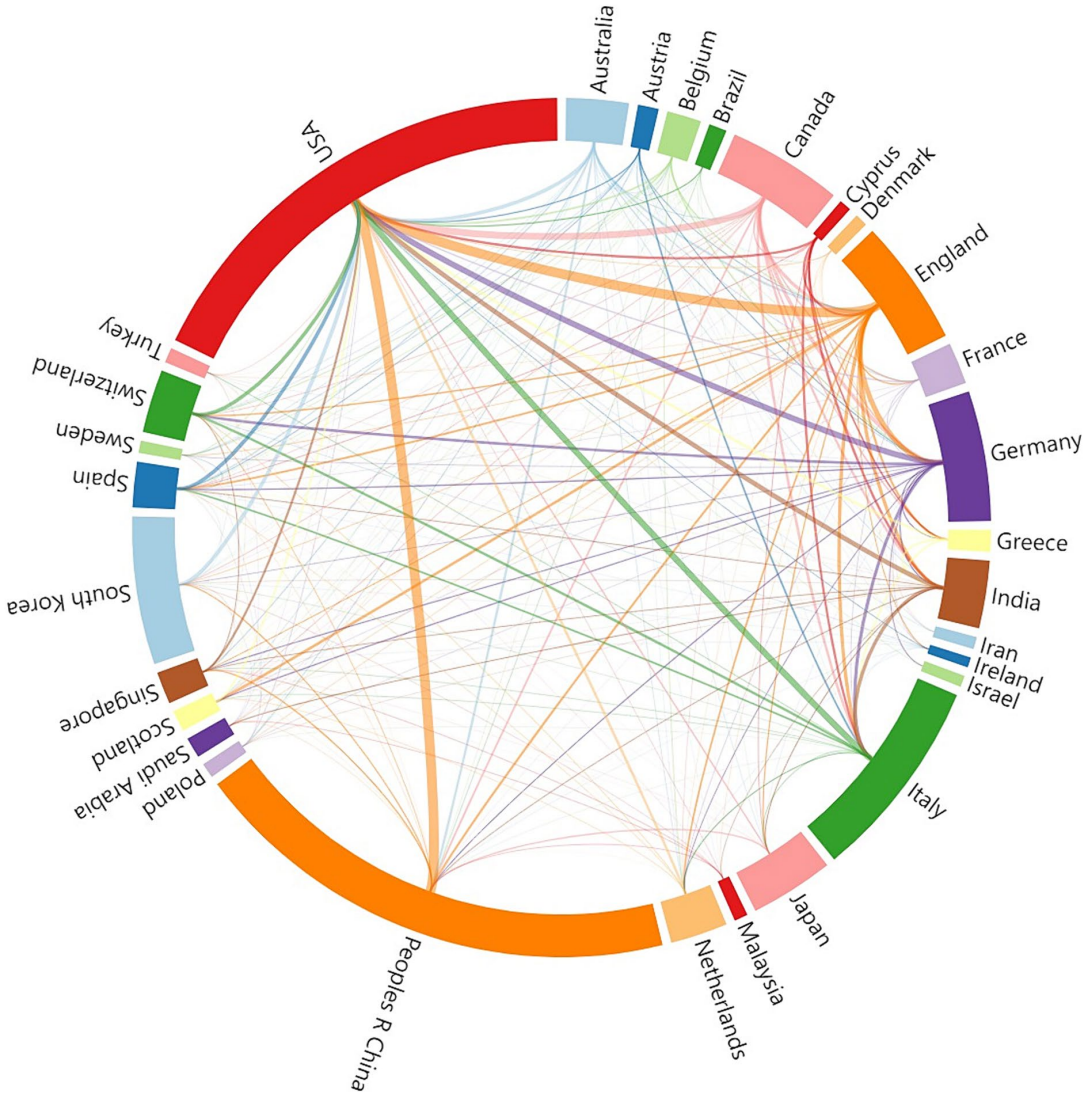
The chord diagram of cooperation between countries/regions about the application of AI in stroke from 2004 to 2024.

A total of 84 countries/regions are involved in AI applications for stroke, and Table 2 displays the top 10 countries/regions in terms of frequency. China was the leading country with 688 articles, followed by the USA (653 articles) and Italy (284 articles). The total link strength (TLS) reflects collaboration intensity with other nations, with the United States demonstrating the highest TLS, signifying robust international cooperation in AI application to stroke. Figure 4 shows a chord diagram of national cooperation, with colored areas representing publications from different countries. The size of each area manifests publication volume, while the line thickness between areas signifies cooperation strength. Notably, the USA exhibits close relations with China, England, and Italy. Moreover, collaboration between other countries still needs further strengthening.

**Table 2:** The top 10 countries/regions in terms of frequency.

Rank	Country/region	Frequency	Total link strength
1	Peoples R China	688	209
2	USA	653	655
3	Italy	284	286
4	South Korea	206	86
5	Germany	181	290
6	England	176	410
7	Canada	160	261
8	Japan	117	66
9	India	94	232
10	Switzerland	88	140

**Figure 5:**
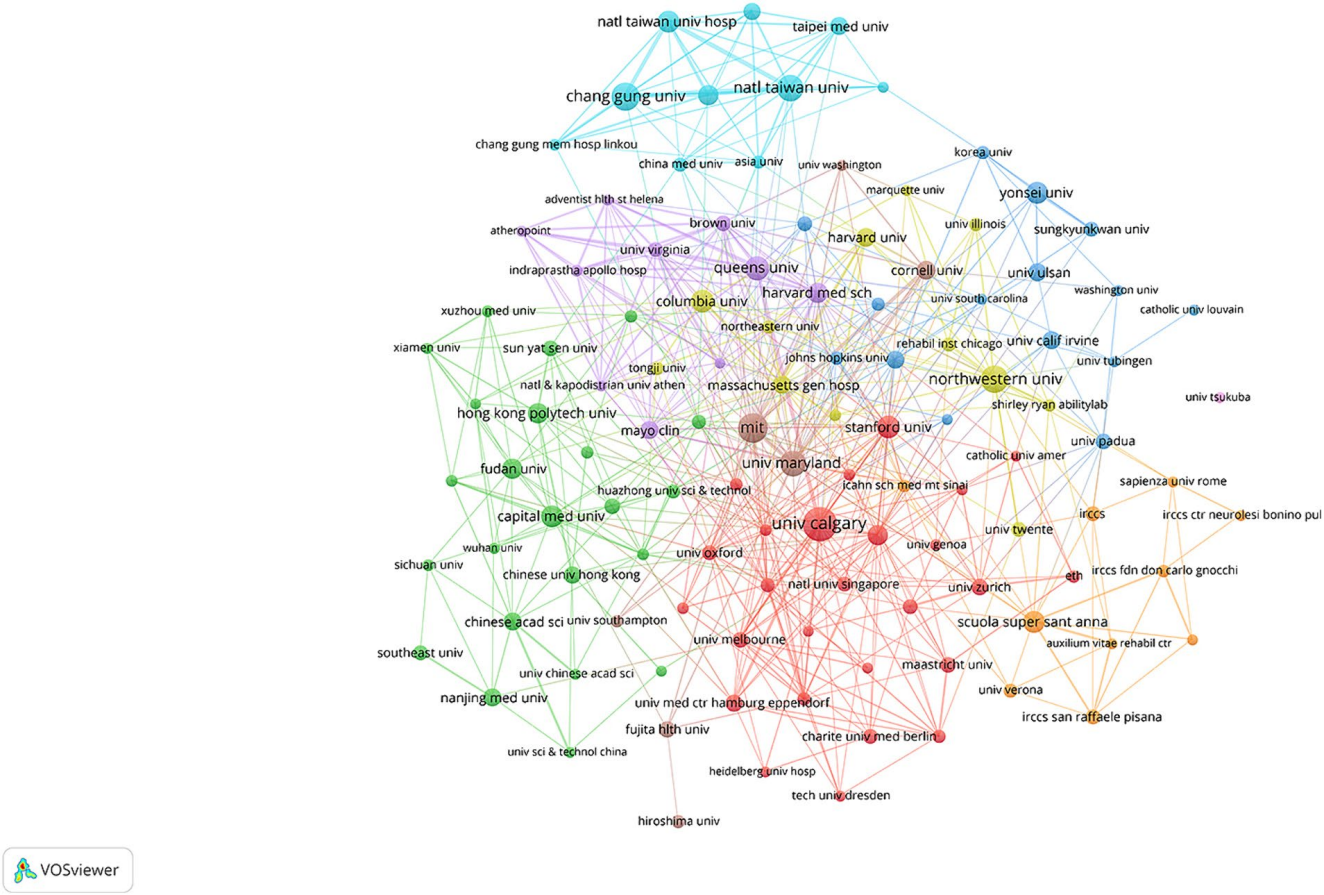
The network visualization diagram of institutional cooperation.

Table 3 outlines the top 10 institutions by publication frequency. The University of Calgary emerges as the most productive organization with 70 papers, while the Massachusetts Institute of Technology boasts the largest TLS. Figure 5 manifests the cooperation network map of institutions, revealing several collaborative clusters headed by the University of Calgary, Capital Medical University, Queen’s University, and National Taiwan University. In addition, we found that cooperation between institutions predominantly occurs between adjacent regions, and academic exchange across different regions requires enhancement.

**Table 3:** The top 10 institutions in terms of frequency.

Rank	Institution	Frequency	Total link strength
1	University of Calgary	70	119
2	Massachusetts Institute of Technology	55	186
3	Chang gung university	51	124
4	Northwestern University	49	78
5	National Taiwan University	47	137
6	University of Maryland	45	153
7	Queen’s University	40	182
8	Stanford University	36	72
9	Columbia University	36	57
10	Capital Medical University	35	56

### 3.4 Analysis of journals and co-cited journals

**Figure 6:**
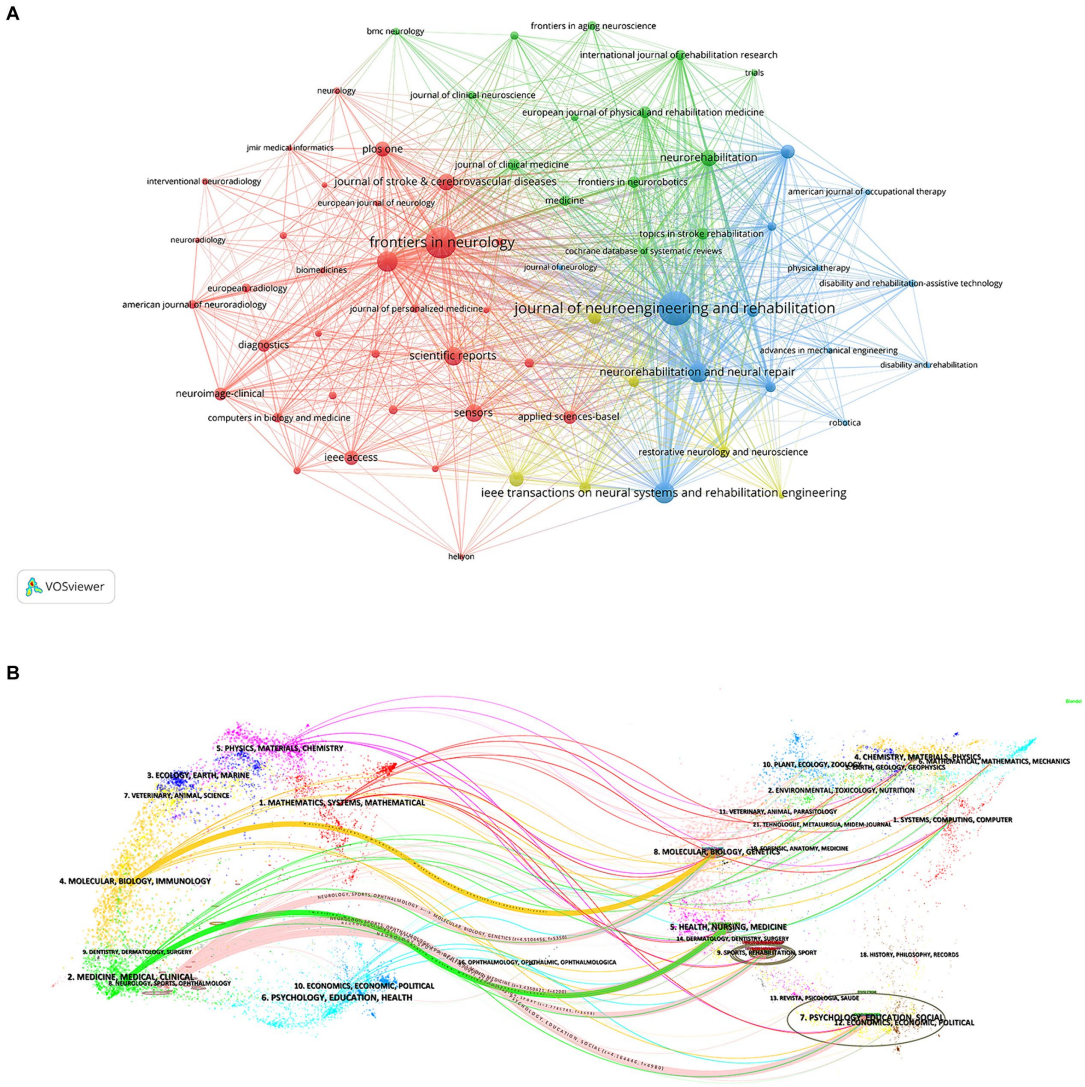
**(A)** The network visualization map of journals; **(B)** The dual-map overlay of journals about the application of AI in stroke.

Tables 4, 5 delineates the top 10 journals and co-cited journals pertaining to the application of AI in stroke. The Journal of Neuroengineering and Rehabilitation leads with the highest number of articles on this topic, followed by Frontiers in Neurology and IEEE Transactions on Neural Systems and Rehabilitation Engineering. The impact factor (IF) of these top 10 journals ranges from 1.7 to 7.8. Figure 6A presents a network visualization map of journals, categorized into different colored clusters. The largest red cluster primarily focuses on neuroimaging, the blue cluster concentrates on rehabilitation, and the green cluster involves neuroscience and neurorehabilitation. Moreover, the journals most frequently co-cited were Stroke (7,925 times), Neurorehabilitation and Neural Repair (3,263 times), and Archives of Physical Medicine and Rehabilitation (3,057 times).

**Table 4:** The top 10 journals by publication frequency about the application of AI in stroke.

Rank	Journal	Counts	2023 JCR IF	2023 JCR partition
1	Journal of Neuroengineering and Rehabilitation	157	5.2	Q1
2	Frontiers in Neurology	131	2.7	Q2
3	IEEE Transactions on Neural Systems and Rehabilitation Engineering	66	4.8	Q1
4	Stroke	62	7.8	Q1
5	Neurorehabilitation and Neural Repair	56	3.7	Q1
6	Scientific Reports	52	3.8	Q1
7	Sensors	47	3.4	Q2
8	Journal of Stroke and Cerebrovascular Diseases	43	2.0	Q3
9	Neurorehabilitation	41	1.7	Q2
10	PLoS One	36	2.9	Q1

**Table 5:** The top 10 co-cited journals by citation frequency about the application of AI in stroke.

Rank	Co-cited journal	Citations	2023 JCR IF	2023 JCR partition
1	Stroke	7,925	7.8	Q1
2	Neurorehabilitation and Neural Repair	3,263	3.7	Q1
3	Archives of Physical Medicine and Rehabilitation	3,057	3.6	Q1
4	Journal of Neuroengineering and Rehabilitation	2,762	5.2	Q1
5	IEEE Transactions on Neural Systems and Rehabilitation Engineering	1,333	4.8	Q1
6	Neurology	1,316	7.7	Q1
7	PLoS One	1,218	2.9	Q1
8	New England Journal of Medicine	1,208	96.2	Q1
9	Neuroimage	1,143	4.7	Q1
10	Lancet	1,077	98.4	Q1

Figure 6B depicts the dual-map overlay of journals. The left side of the map shows citing journals, indicating the frontiers of knowledge, while the right side displays cited journals, representing the foundation of knowledge. Colored paths denote citation links, delineating the association between various research disciplines. The most prominent pink citation lines manifest that studies in the fields of molecular/biology/genetics, health/nursing/medicine, sports/rehabilitation/sport, and psychology/education/social are frequently cited by research in neurology/sports/ophthalmology.

### 3.5 Analysis of keywords

**Figure 7:**
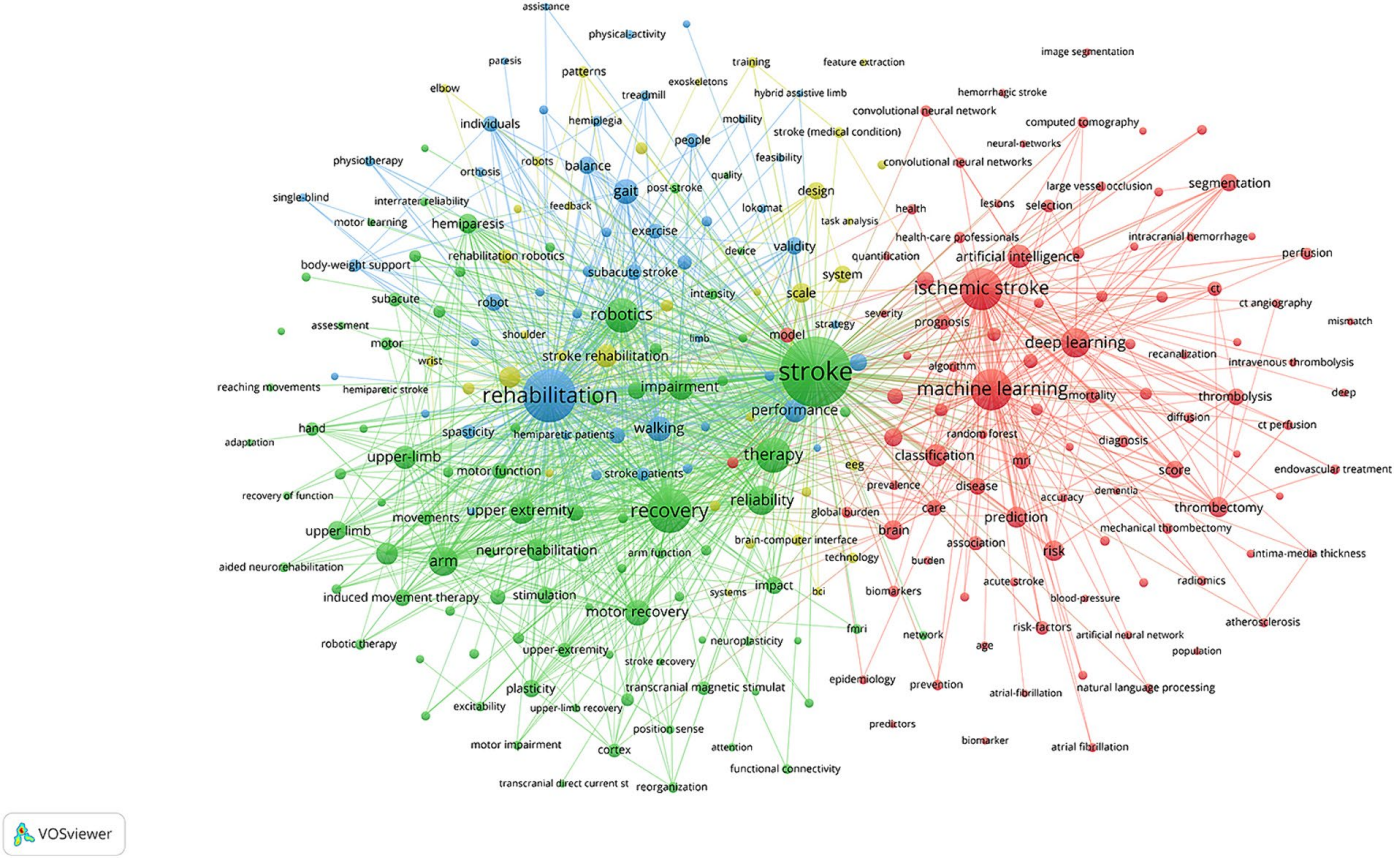
The clustering network map of keywords.

**Figure 8:**
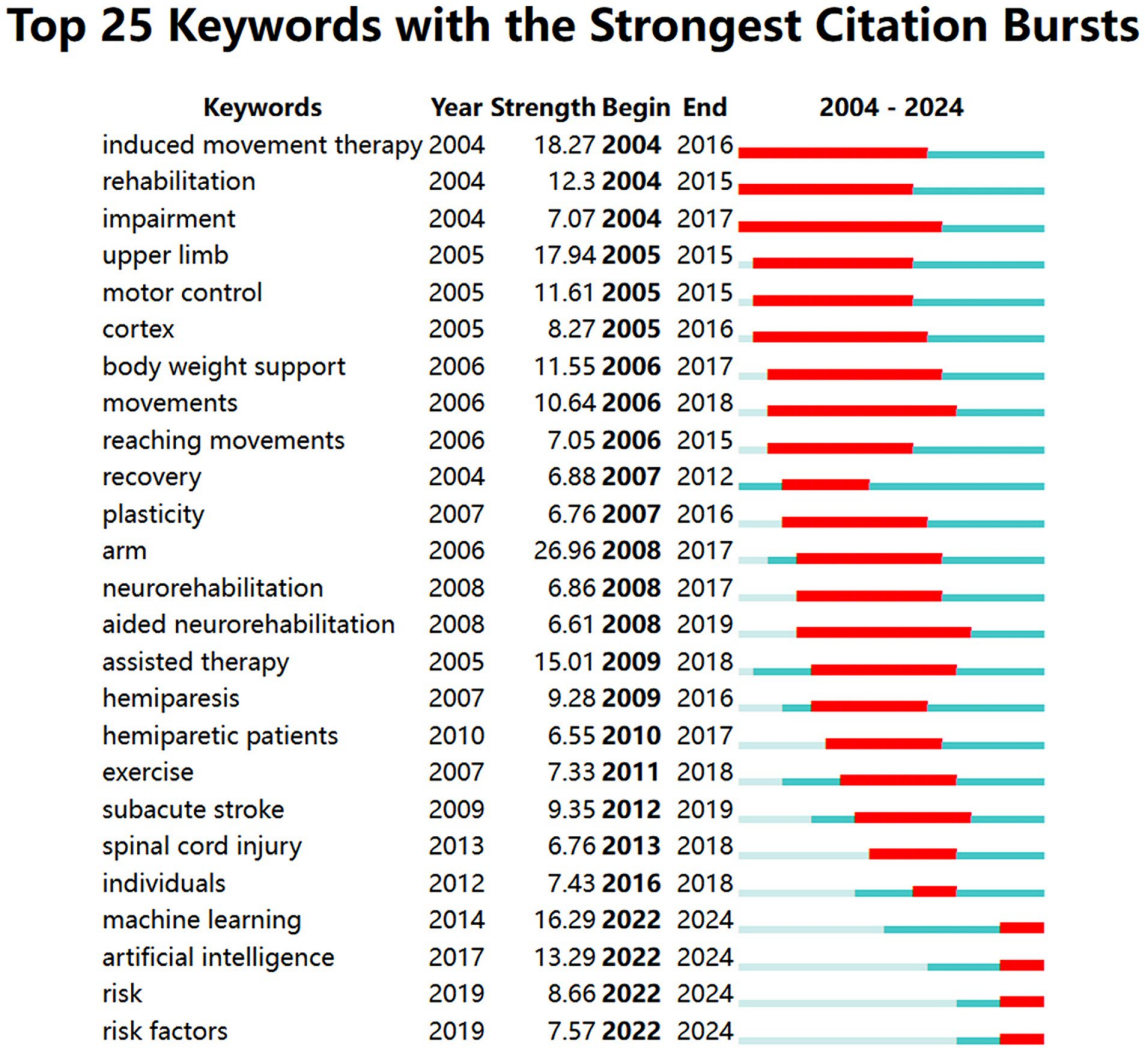
The top 25 keywords with citation bursts about the application of AI in stroke.

After analyzing keywords with a frequency of more than 15 times, Figure 7 displays a network visualization map of keywords. Notably, there are three prominent clusters, which mainly encompass stroke, machine learning, and rehabilitation. Additionally, Figure 8, generated by CiteSpace, shows the top 25 keywords with the strongest citation bursts, facilitating the exploration of research focus and frontiers. Burst strength and time span serve as significant indicators in keyword burst analysis. The top three keywords with the strongest citation burst are “arm” (2008–2017), “induced movement therapy” (2004–2016), and “upper limb” (2005–2015). Examining keyword bursts in recent years aids in predicting future research trends and topics. Hence, keywords such as “artificial intelligence,” “machine learning,” “risk,” and “risk factors” offer valuable references for future studies.

### 3.6 Analysis of co-cited references

Co-citation analysis of references offers insights into the knowledge foundation of the research field and identifies influential literature. The top 10 co-cited references in terms of citation counts are shown in Table 6, among which a retrospective study by Heo J. in 2019 was the most frequently cited. This study, involving 2,604 patients, demonstrated that machine learning models contribute to predicting long-term outcomes in acute stroke patients. Notably, the deep neural network model performed better than the random forest and logistic regression models (Heo et al., 2019).

**Table 6:** The top 10 co-cited references about the application of AI in stroke.

Rank	Co-cited references	References	Counts
1	Machine Learning-Based Model for Prediction of Outcomes in Acute Stroke	Heo et al. (2019)	105
2	Thrombectomy for Stroke at 6–16 h with Selection by Perfusion Imaging	Albers et al. (2018)	91
3	Thrombectomy 6–24 h after Stroke with a Mismatch between Deficit and Infarct	Nogueira et al. (2018)	88
4	Effects of Robot-Assisted Therapy for the Upper Limb After Stroke	Veerbeek et al. (2017)	86
5	Effects of robot-assisted therapy on upper limb recovery after stroke: a systematic review	Kwakkel et al. (2008)	69
6	Robot assisted training for the upper limb after stroke (RATULS): a multicenter randomized controlled trial	Rodgers et al. (2019)	68
7	Guidelines for the Early Management of Patients with Acute Ischemic Stroke: 2019 Update to the 2018 Guidelines for the Early Management of Acute Ischemic Stroke: A Guideline for Healthcare Professionals from the American Heart Association/American Stroke Association	Powers et al. (2019)	67
8	2018 Guidelines for the Early Management of Patients with Acute Ischemic Stroke: A Guideline for Healthcare Professionals from the American Heart Association/American Stroke Association	Powers et al. (2018)	61
9	Prediction of Tissue Outcome and Assessment of Treatment Effect in Acute Ischemic Stroke Using Deep Learning	Nielsen et al. (2018)	54
10	Artificial intelligence to diagnose ischemic stroke and identify large vessel occlusions: a systematic review	Murray et al. (2020)	54

**Figure 9:**
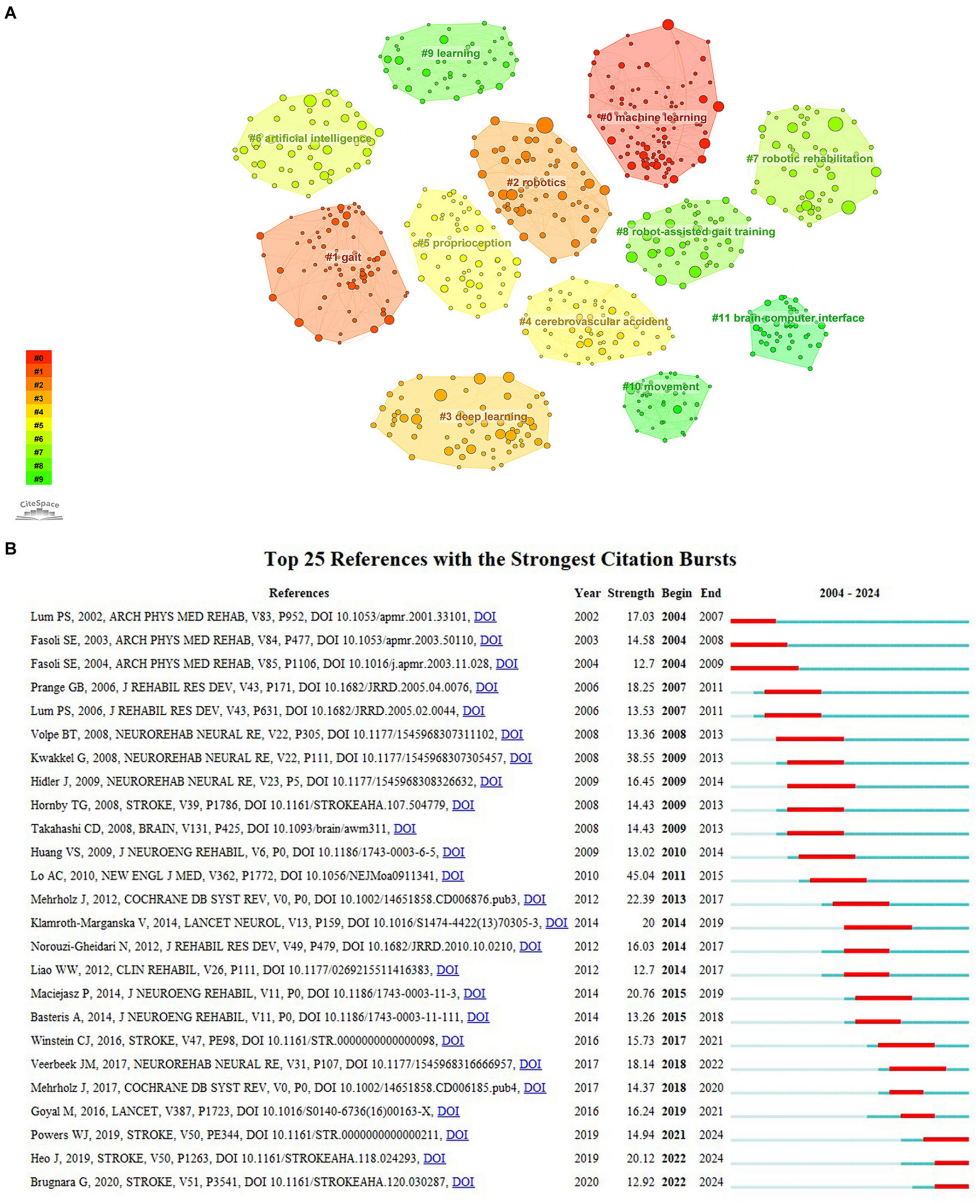
**(A)** The clustering analysis of co-cited references; **(B)** The top 25 references with citation bursts about the application of AI in stroke.

Clustering analysis of references applying CiteSpace generated 100 clusters with modularity and weighted mean silhouette values of 0.8705 and 0.9468, respectively. Figure 9A depicts the top 12 clusters, with the three largest clusters focusing on machine learning, gait, and robotics. Besides, deep learning, robotic rehabilitation, robot-assisted gait training, and brain-computer interfaces are also considered research emphases.

Reference bursts manifest a significant increase in the attention of articles within a certain period of time. As shown in Figure 9B, the publication with the highest burst strength (45.04) among the top 25 co-cited references is “Robot-assisted therapy for long-term upper-limb impairment after stroke.” This article revealed that robot-assisted rehabilitation for 12 weeks did not show significant advantages compared to usual care and intensive comparative treatment in patients with moderate to severe upper-limb impairment 6 months post-stroke. Nevertheless, over 36 weeks, robot-assisted treatment significantly improved the motor function of patients compared to usual care, but not compared to intensive comparative treatment (Lo et al., 2010). Furthermore, a systematic review of robot-assisted therapy for upper limb recovery post-stroke has gained the second-largest burst strength from 2009 to 2013. It highlighted the potential of robot-assisted therapy for enhancing proximal upper extremity function (Kwakkel et al., 2008). The citation bursts of the references in the lower three lines of Figure 9B extend until 2024, including a guideline on the management of acute ischemic stroke (Powers et al., 2019) and two studies on using machine learning to predict clinical outcomes in stroke patients (Heo et al., 2019; Brugnara et al., 2020). These provide information for identifying research frontiers in AI applications for stroke.

## 4 Discussion

### 4.1 Summary of basic information

In this study, we conducted a bibliometric analysis of relevant literature concerning the application of AI in stroke retrieved from 2004 to 2024. A total of 2,447 articles were included, with a peak of 462 publications in 2023. The annual publication count remained below 100 until 2017, but it has grown rapidly after 2019. According to Table 1, the authors with the highest publications and the largest average citation are Dukelow, Sean P., and Scott, Stephen H., respectively. Their work appears to be highly influential and contributes to the development of this field. Notably, several stable collaborative groups of authors and institutions have emerged, although there remains a need to further strengthen cross-regional academic cooperation. In terms of countries, China ranks first with 688 articles and shows close cooperation with the United States. The Journal of Neuroengineering and Rehabilitation, Stroke, and Neurorehabilitation and Neural Repair appeared in the top 10 journals and co-cited journals in terms of frequency, serving as valuable references for future scholars in this field.

### 4.2 Research hotspots and emerging trends

#### 4.2.1 Machine learning

According to Figures 8, 9A, the largest cluster, labeled #0 machine learning, not only represents a current research hotspot but also signifies the forefront of future investigations. Machine learning (ML) stands as an important subset of AI, wherein algorithms learn from data without explicit programming. ML encompasses techniques such as linear regression, logistic regression, support vector machines, and decision trees (Mouridsen et al., 2020). It was extensively employed to determine and predict stroke onset time, enhance the efficiency and accuracy of stroke diagnosis, and predict clinical outcomes and risks (Amukotuwa et al., 2019; Heo et al., 2019; Feng et al., 2023; Hong et al., 2023). Cluster #3, deep learning (DL), is an ML approach based on artificial neural networks with multiple layers to learn complex patterns presented in the input data (Karakis et al., 2023). There are two main subtypes of neural networks, among which recurrent neural networks connect nodes that form a directed graph along a time series, while convolutional neural networks calculate spatial relationships among different pixel regions in an image. Various DL methods have been developed for the detection of acute ischemic stroke, intracranial hemorrhage (ICH), and its subtypes (Chilamkurthy et al., 2018; Meijs et al., 2020). Lesion segmentation and quantitative analysis of images are crucial for stroke diagnosis, evaluation, and treatment (Chavva et al., 2022). Clèrigues et al. (2020) illustrated that a 3D patch-based DL approach using the U-Net architecture yielded superior lesion segmentation, facilitating quick quantitative estimation of the extent and location of the penumbra area. Therefore, ML and DL play an increasingly significant role in stroke management with relatively high efficiency, reliability, and repeatability.

#### 4.2.2 The application of AI in stroke imaging for early identification and diagnosis

Neuroimaging is widely used in stroke research, and the common preferred examination for suspected stroke patients is computed tomography (CT) scanning. It identifies cerebral hemorrhage or ischemia and evaluates the extent and severity of lesions (Sarmento et al., 2020). However, interpreting subtle image changes often relies on the expertise of radiologists, which can vary among practitioners. To address this variability, AI has been applied to quickly and accurately analyze imaging results (Ding et al., 2020). A study processed CT angiography on 477 patients using an automatic detection algorithm for large vessel occlusion, achieving a high diagnostic sensitivity of 94% and a negative predictive value of 98% within 5 min (Amukotuwa et al., 2019). AI enhanced diagnostic efficiency, particularly for patients requiring transfer to comprehensive stroke centers for thrombectomy, thereby mitigating further brain damage and improving prognosis (Leslie-Mazwi and Lev, 2020). The Alberta Stroke Program Early CT Score (ASPECTS) is an approach for assessing the severity of acute ischemic stroke by employing non-contrast CT (Pacchiano et al., 2024). Maegerlein et al. (2019) indicated that ASPECTS assessments calculated by ML-based automatic software tools exhibit greater consistency with predefined consensus standards than those of experienced neuroradiologists in cases of middle cerebral artery occlusion. Furthermore, the software performed better in detecting infarction regions at a 1–4 h interval between symptom onset and imaging, underscoring its potential for early intervention. In summary, AI applications facilitate more prompt and sensitive stroke identification to guide clinical decision-making and improve care.

#### 4.2.3 Stroke rehabilitation using AI

The primary neurological impairment following a stroke is hemiplegia, which affects the patient’s ability to engage in daily activities and causes inconvenience. Rehabilitation plays a pivotal role in stroke management, facilitating the recovery of impaired functions and enhancing overall quality of life (Hwang and Song, 2023). Cluster #7 Robotic rehabilitation is a research hotspot that encompasses assistive robots and exoskeletons (Rahman et al., 2023). A recent meta-analysis highlighted the significant enhancement of upper extremity motor function and activities of daily living in stroke patients undergoing robot-assisted rehabilitation training (Yang X. et al., 2023). However, a study published in Lancet in 2019 revealed no significant improvements in patients with moderate or severe upper limb functional impairment after stroke when compared with routine care (Rodgers et al., 2019). This may result from potential disparities in patient characteristics and treatment protocols. Singh et al. (2021) demonstrated that robotic hand exoskeleton training improved motor ability and enhanced cortical excitability of the ipsilesional hemisphere in stroke patients, which may be attributed to plastic reorganization and use-dependent plasticity.

Notably, approximately one-third of stroke survivors fail to regain the ability to walk independently, underscoring the significance of the rehabilitation of post-stroke gait impairment (Benjamin et al., 2018; Calabrò et al., 2021). Cluster #8 Robot-assisted gait training is constantly evolving, aiming to enhance limb coordination and neuroplasticity through specific repetitive motor coordination exercises. Studies have indicated the effectiveness of robotic exoskeletons in ameliorating gait disorders among both subacute and chronic stroke survivors (Calafiore et al., 2022; Yang J. et al., 2023). Cluster #11 brain-computer interface (BCI) technology, which directly connects the human brain and external devices, has shown promise in stroke rehabilitation. Zhao et al. (2022) found that 4 weeks of BCI-controlled robot training in subacute stroke patients led to the recovery of motor function in the lower extremities, increased serum BDNF levels, and improved cognitive function. In conclusion, AI has posed great potential in stroke rehabilitation. Further clinical trials are warranted to validate its efficacy and stability, with the ultimate goal of developing personalized and optimized rehabilitation plans.

#### 4.2.4 AI for outcomes and risk prediction of stroke

Through the analysis of citation bursts in keywords and references, we found that stroke outcomes and risk prediction may be emerging research trends in this field. In clinical practice, physicians are often required to provide estimates of patient mortality, the incidence of complications, and the degree of functional dependence to guide care and treatment (Mainali et al., 2021). A retrospective study of 11,775 patients from the Swedish Stroke Register, utilizing logistic regression, revealed that 75.2% of stroke patients had comorbidities, such as atrial fibrillation and hypertension. High comorbidities (≥4) increased mortality rates from 27.3% at 12 months post-stroke to 64.6% at 5 years post-stroke. And patients without comorbidities had mortality rates of 7.3% at 12 months and 19.4% at 5 years post-stroke (Sennfält et al., 2020). After reviewing the prognostic scores of patients with acute ischemic stroke, Matsumoto et al. found that ensemble models of decision trees outperformed linear regression models in predicting adverse functional outcomes (Matsumoto et al., 2020). In terms of ICH, a study utilized machine learning algorithms to evaluate CT imaging in patients with spontaneous ICH, demonstrating high accuracy in predicting functional outcomes (evaluated by the modified Rankin Scale). Importantly, the addition of the ICH score improved predictive efficacy (Nawabi et al., 2021).

Guidelines recommend the use of risk predictive tools to promote stroke prevention, screening, and therapeutic interventions (Meschia et al., 2014). The original Framingham Stroke Risk Profile (FSRP) predicted 10-year stroke risk based on crucial risk factors identified in epidemiological studies. Since then, various predictive models have been developed, such as the QStroke algorithm and the revised FSRP (Wolf et al., 1991; Hippisley-Cox et al., 2013; Dufouil et al., 2017). Lip et al., 2022 conducted a study on a prospective US cohort of 3,435,224 patients, demonstrating that ML-based algorithms could predict stroke risk by analyzing complex relationships among various comorbidities, both cardiovascular and non-cardiovascular. It also provided automated methods for dynamic risk stratification. Given population differences, 10-year stroke risk equations were introduced to assess both 10-year and lifetime stroke risk in Chinese adults, showing superior predictive capability compared to the revised FSRP (Xing et al., 2019). Another study identified age, sex, hypertension, and low-density lipoprotein cholesterol as risk predictors for ischemic stroke, while age, sex, hypertension, body mass index, and high-density lipoprotein cholesterol as important risk factors for hemorrhagic stroke (Yu et al., 2021). As AI technology advances, future research should aim to include diverse populations from broader databases and explore additional predictive factors to enhance prediction accuracy, prevent stroke occurrence and progression, and improve patient prognosis.

### 4.3 Limitations

Although we have conducted a thorough and systematic bibliometric analysis of the application of AI in stroke, certain limitations exist. Firstly, Web of Science, a highly influential database, serves as a vital resource for academic analysis. It provides information for citation analysis and enables a deeper exploration of the research landscape within the field. However, we only employed this database for analysis, potentially neglecting valuable literature in other databases. Secondly, due to language restrictions, we only analyzed articles in English, and articles in other languages were not included in this analysis.

## 5 Conclusion

To our knowledge, this study represents the first comprehensive bibliometric analysis of publications from the past two decades that delves into the knowledge structure and development trajectory of AI applications in stroke. Our findings reveal a notable surge in annual publications, indicating an increasing interest in this topic. The most prolific authors, countries, and institutions are Dukelow, Sean P., China, and the University of Calgary, respectively, making substantial contributions to the advancements of this field. However, there remains a need for enhanced cross-regional and international collaboration to further bolster progress. The application of AI in stroke involves the intersection of multiple disciplines. Therefore, it is necessary to better integrate expertise across various fields to drive rapid advancements in stroke management. Our analysis highlights predominant research hotspots, which include machine learning, deep learning, and AI’s role in stroke rehabilitation and imaging for early identification and diagnosis. Moreover, emerging trends focus on machine learning as well as stroke outcomes and risk prediction. It is foreseeable that AI will increasingly play a crucial role in stroke prevention, treatment, and prognosis. This development is advantageous for optimizing the utilization of medical resources and enhancing the quality of life for patients.

## Data Availability

The original contributions presented in the study are included in the article/supplementary material, further inquiries can be directed to the corresponding author.
